# Exposing current tobacco industry lobbying, contributions, meals, and gifts

**DOI:** 10.18332/tid/144765

**Published:** 2022-01-21

**Authors:** Beth Rotman, Gabrielle Ballweg, Nichelle Gray

**Affiliations:** 1 Common Cause, Washington, United States; 2 Action on Smoking and Health, Washington, United States

**Keywords:** tobacco industry lobbying, tobacco industry, smoking, public health

Informed by internal tobacco industry documents revealed through state-level lawsuits, a federal court ruled in 2006 that Altria, R.J. Reynolds, and other tobacco companies, had repeatedly violated the Racketeer Influenced and Corrupt Organizations (RICO) Act^1^. The 1682-page ruling identified 145 distinct acts of racketeering, concluding that they ‘cannot be trusted with the responsibility of identifying and implementing the necessary changes in their own companies’, that they ‘have not ceased engaging in unlawful activity’, and would likely continue to commit fraud ‘indefinitely into the future’. After 11 years of appeals, the companies began publishing court-ordered corrective statements in November 2017^2-4^. The RICO case remains active, with the companies still opposing placement of the statements at retail points-of-sale^5^.

Public records and eyewitness reports indicate that tobacco companies continue to employ tactics detailed in their internal documents at state capitols across the country^6^. Such tactics include creating and spreading disinformation; hiring influential lobbyists and lobbying firms; requiring their lobbyists to seek corporate review and approval of significant activities; donating directly to political campaigns while empowering their lobbyists to donate contributions, meals and gifts in their own names; building and nurturing strategic alliances with front groups; and proactively seeking legislation of their own design (e.g. state preemption of local tobacco ordinances) ^7^.

A quote from a 1992 Philip Morris document explained that ‘public opinion and media coverage are only important insofar as they affect the government – we will never be liked and what we want is to be ignored’^8^. This aversion to publicity typifies the tobacco industry’s strong preference to work behind the scenes when seeking to influence policy^9^. There are at least four reasons why public health leaders should shine a light on tobacco industry interference in lawmaking. First, tobacco industry behaviors have caused smoking to become the world’s top cause of preventable death^10^. Second, the most effective tools for preventing tobacco use involve public policy^11^. Third, the greatest barrier to enacting effective tobacco prevention policy is tobacco industry interference^12^. Fourth, publicly exposing tobacco industry interference serves to reduce its detrimental influences and long-lasting effects on public health^13-15^.

Media advocacy is recognized as an essential practice in tobacco control^16^. Over the years, researchers and advocates have used public records to illuminate tobacco industry interference at the state level^17^. The website tobaccomoney.com was launched in 2012 to expose historical and ongoing tobacco industry interference in the Oklahoma State Legislature. The site listed registered tobacco lobbyists by name, key findings from the RICO case, and Oklahoma-specific quotes from internal documents. This information was reported by numerous local media outlets, augmenting their coverage of tobacco policy issues^18^. The RICO verdict and the current number of registered tobacco lobbyists have become part of the public narrative on tobacco policy issues in that state^18,19^.

Inspired by the initiative in Oklahoma, Action on Smoking and Health (ASH) recently launched the U.S. Tobacco Lobbyist and Lobbying Firm Registration Tracker. This tracker exposes tobacco industry lobbyists and lobbying firms across all 50 states and the District of Columbia (D.C.). The research was conducted on publicly available state-level registrations from July through October 2021 and consisted of visiting the lobbying registration websites for all 50 states and D.C. These public records were searched for tobacco companies, tobacco industry trade associations, and tobacco retailers currently employing lobbyists or lobbying firms.

In 2021, 994 state-level lobbying registrations for the tobacco industry were identified, involving at least 918 individual lobbyists or lobbying firms, some of whom registered to represent more than one tobacco company, association, or outlet. Of these registrations, about two-thirds (639) represented a company owned wholly or in part by federally adjudicated racketeers, including Altria and Reynolds American. These tobacco companies were charged after conspiring to conceal the health risks and the addictiveness of cigarettes. Many tobacco industry lobbyists who officially represent companies, associations, or outlets that are not specifically part of the RICO case still work closely with these racketeers^20^. Therefore, policymakers should never trust information provided by lobbyists who work for federally adjudicated racketeers or their allies.

Of the 994 total state-level lobbying registrations for the tobacco industry, Altria has the greatest number of registrations with 300 registered lobbyists or lobbying firms representing Altria’s interests across all 50 states and D.C. JUUL, of which Altria owns a 35% stake, employed a total of 138 registered lobbyists or lobbying firms covering 49 states and D.C., and Reynolds American registered a total of 201 lobbyists or lobbying firms covering 49 states. Most tobacco industry lobbyists and lobbying firms also serve a variety of other clients including schools or health groups, presenting serious conflicts of interest.

Furthermore, lobbying registration laws are inconsistent across states. The tobacco industry leverages loopholes in lobbying registration laws to mask from public view the actual number and names of all of the individual lobbyists who are working on their behalf. In 13 states and D.C., it was impossible to determine from online records the total number of lobbyists within a single registered lobbying firm who perform work on behalf of the tobacco industry. Therefore, the actual number of tobacco industry lobbyists in these states and D.C. is likely much greater than these data suggest. For more information about an individual state, please visit ASH’s U.S. Tobacco Lobbyist and Lobbying Firm Registration Tracker.

Tracking and exposing tobacco industry interference in the United States will continue to be necessary until Congress enacts systemic reforms. Permanently fixing the problem of Big Tobacco requires fixing our democracy. Politics costs a lot of money. Runaway campaign spending blocks better government policies because candidates turn to the wealthy and industry for support. This support comes with strings attached because big spenders are investing in policy outcomes. Wealthy special interests like Big Tobacco are all too happy to fund candidates in their push for outcomes. And Big Tobacco can afford high priced lobbying efforts in addition to direct contributions to candidates.

The powerful tobacco lobby will seemingly spend whatever it takes to keep politicians beholden to them and maintain a toxic *status quo*. This vicious cycle, essentially a takeover by Big Money, blocks many government policies favored by voters. So how do we finally get Big Money out of our democracy and make room for better policies that favor everyday Americans?

A good starting point can be found in the ‘Freedom to Vote’ Act. This Act closes loopholes that favor big corporations and the wealthy, makes it easier for all people to vote, and strengthens the power of small donors through public financing of elections: a system that empowers all Americans and not just the wealthy few.

Democracy is how we tackle the extraordinary challenges facing our country and agree on solutions. The influence of Big Money in politics and conflicts of interest skew the playing field of our democracy and interfere with attaining the best solutions. However, ‘the many’ can overcome ‘the money’, and Common Cause is working on this state-by-state, and at a national level^21^. No matter our race, background or zip code, most of us believe that for democracy to work for all of us, it must include us all. When Big Tobacco and Big Money win, Americans lose.

## Data Availability

Data sharing is not applicable to this article as no new data were created.
